# A novel approach combining self-organizing map and parallel factor analysis for monitoring water quality of watersheds under non-point source pollution

**DOI:** 10.1038/srep16079

**Published:** 2015-11-03

**Authors:** Yixiang Zhang, Xinqiang Liang, Zhibo Wang, Lixian Xu

**Affiliations:** 1 College of Environmental and Resource Sciences, Zhejiang University, Hangzhou 310058, China; 2 Zhejiang Provincial Key Laboratory for Water Pollution Control and Environmental Safety.

## Abstract

High content of organic matter in the downstream of watersheds underscored the severity of non-point source (NPS) pollution. The major objectives of this study were to characterize and quantify dissolved organic matter (DOM) in watersheds affected by NPS pollution, and to apply self-organizing map (SOM) and parallel factor analysis (PARAFAC) to assess fluorescence properties as proxy indicators for NPS pollution and labor-intensive routine water quality indicators. Water from upstreams and downstreams was sampled to measure dissolved organic carbon (DOC) concentrations and excitation-emission matrix (EEM). Five fluorescence components were modeled with PARAFAC. The regression analysis between PARAFAC intensities (*F*_max_) and raw EEM measurements indicated that several raw fluorescence measurements at target excitation-emission wavelength region could provide similar DOM information to massive EEM measurements combined with PARAFAC. Regression analysis between DOC concentration and raw EEM measurements suggested that some regions in raw EEM could be used as surrogates for labor-intensive routine indicators. SOM can be used to visualize the occurrence of pollution. Relationship between DOC concentration and PARAFAC components analyzed with SOM suggested that PARAFAC component 2 might be the major part of bulk DOC and could be recognized as a proxy indicator to predict the DOC concentration.

Agricultural and rural non-point source (NPS) pollution is mainly caused by the release of fertilizers, pesticides and other additives applied in agricultural lands^1^. Rainfall and irrigation are the major drivers of the loads of agricultural NPS pollution, and runoff is the carrier to transport contaminants and decides the composition and quantity of the pollution^2^. A diversity of land use, a wide range of inputs, a variety of release mechanisms and pathways and other complex factors, contribute to the uncertainty, randomness, complexity, intermittence and variability of agricultural NPS pollution^3^. The sources of NPS pollution include natural origin (*e.g.* soils, crops and microorganisms) and anthropogenic origin (fertilizers and pesticides). The agricultural and rural NPS pollution mainly includes: (1) nutrient elements such as nitrogen and phosphorus caused by high rates of fertilization, which lead to eutrophication in ambient waters^4^; (2) organic matters derived from soils, fertilizers and/or pesticides, which lead to uncomfortable concerns like color, taste and odor, bring about rise of organic pollution indicators (*e.g.* chemical oxygen demand (COD)), create toxicity in aquatic ecosystems (*e.g.* pesticides), introduce emerging organic contaminants (*e.g.* pharmaceutical and personal care products (PPCPs) such as hormones and antibiotic resistance genes derived from manure fertilization)^5^, and increase the risk of disinfection byproducts (DBPs) formation (dissolved organic matter (DOM) is the precursors of DBPs)^6^; (3) pathogens derived from manure fertilization^7^.

DOM is a kind of mixture which is so far still poorly defined. DOM can be classified into two categories according to origin: (1) allochthonous DOM which is terrestrially derived and dominated by humic substances; (2) autochthonous DOM which is microbially derived and dominated by non-humic organic matter^6^. Allochthonous sources include soil organic matter, plants and dissolved atmospheric dust, which is characteristic by high aromacity, high molecular weight and low nitrogen content. Autochthonous sources include microorganisms, algae and macrophytes, which is characteristic by low aromacity, low molecular weight and high nitrogen content. DOM can also be fractionated into several categories according to physical and/or chemical characteristics, for example, XAD resin adsorption, ultrafiltration (UF) and size exclusion chromatography (SEC)^8^. The application of fluorescence excitation-emission matrix (EEM) provides a new approach to achieve knowledge about DOM composition. Several methods have been developed to analyze information and extract fluorophores from EEM spectroscopy: (1) peak-picking techniques which extract several basic and significant model fluorescence peak^9^,^10^; (2) fluorescence regional integration (FRI) technique which integrate fluorescence intensity values in five divided excitation-emission regions^11^; (3) principal component analysis (PCA) which extract principal compositions from EEM^12^; (4) parallel factor analysis (PARAFAC) which is a supervised algorithm to decompose DOM fluorescence into components with an optimal number^13^; (5) self-organizing map (SOM) which is an unsupervised algorithm for fluorescence data decomposition and pattern recognition^14^; (6) approaches combining methods above (*e.g.* combination of PARAFAC and SOM)^15^,^16^.

EEM has been considered as a competitive analytical tool applied to examine water quality in natural and engineering aquatic systems. In water supply systems, EEM was used as an assessment approach for water quality from groundwater systems^13^, surface water systems^17^, and recycled water systems^18^,^19^,^20^. In wastewater treatment systems, EEM was used as a technique to evaluate removal efficiency of organic matter from a typical wastewater treatment plant^21^, reverse osmosis systems^22^, swimming pools^23^. In natural water systems, EEM was used a monitoring tool for river pollution from sewerage^24^, soils and plant material^25^, and urban pollution^26^,^27^.

The objectives of this study were to (1) characterize and quantify DOM in a watershed affected by NPS pollution, (2) assess fluorescence properties with SOM analysis as proxy indicators of NPS pollution, and (3) assess the accuracy and reliability of capturing DOM components by monitoring raw fluorescence at a small number of target wavelengths rather than massive EEM measurements.

## Results and Discussion

### Fluorescence characterization of DOM

**Figure 1:**
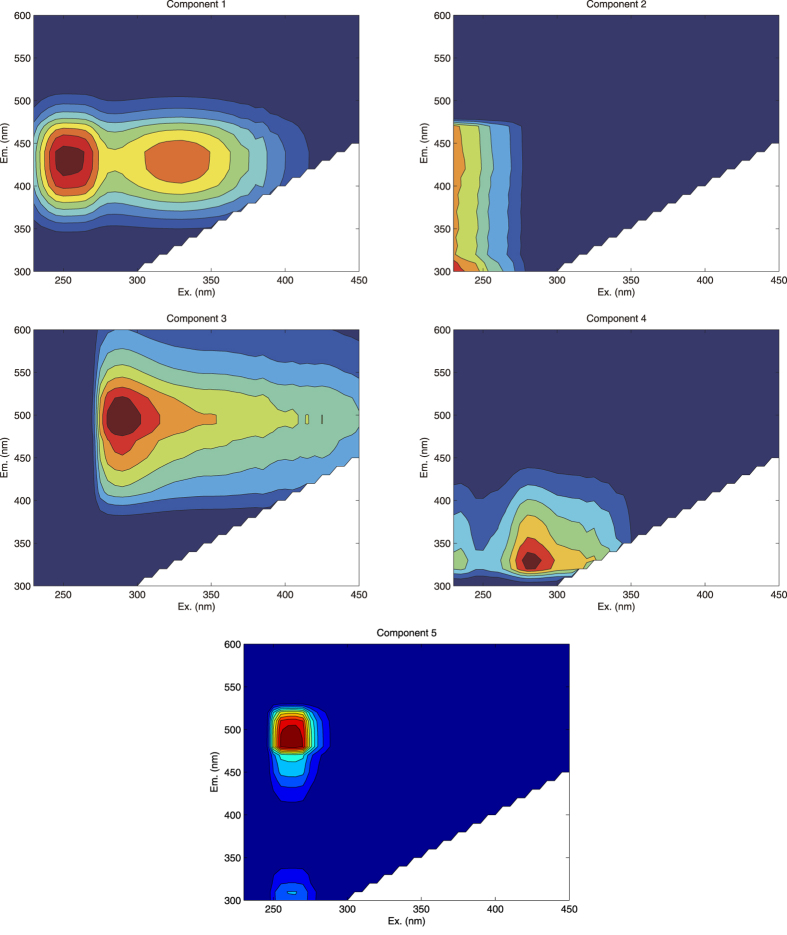
The spectral characteristics of the five fluorescence components identified by the PARAFAC model. The figures were created using MATLAB 7.0.

PARAFAC is considered as a robust analytical tool to discriminate DOM compositions from massive data of EEMs^20^,^21^. A five-component model was developed to explain the majority of fluorescence information from EEMs. Figure 1 shows the modeled component spectra of the five components. Component 1 had a peak at *λ*_ex_/*λ*_em_ = 250/440 nm and a shoulder at *λ*_ex_/*λ*_em_ = 330/440 nm. Fluorescence in this region is referred to as peak A (humic-like) based on Coble^9^,^10^ or as Region III (fulvic acid-like) based on FRI technique by Chen, *et al.*^11^. Fulvic-like DOM is ubiquitous in natural water. Component 2 had a peak at *λ*_ex_/*λ*_em_ = 230/300 nm, whose shape was different from component 1. It overlaps with the region of peak B (tyrosine-like) based on Coble^9^,^10^ and Region I (aromatic protein) based on FRI technique by Chen, *et al.*^11^ (2003). This type of DOM composition has been observed in biological processes during bloom periods^10^. Component 3 had a similar fluorescence shape to component 1 with a peak at *λ*_ex_/*λ*_em_ = 290/490 nm. Fluorescence of component 3 had a similar location to peak C (humic-like) based on Coble^9^,^10^ and fell into Region V (humic acid-like) based on FRI by Chen, *et al.*^11^. Component 4 had a similar spectral characteristics to that of peak T_1_ (tryptophan-like) with the peak at *λ*_ex_/*λ*_em_ = 280/330 nm and a shoulder at *λ*_ex_/*λ*_em_ = 235/330 nm. The majority of component 4 located in Region IV is considered as soluble microbial product (SMP)-like by Chen, *et al.*^11^, which is frequently observed in waterways impacted by wastewater treatment plant (WWTP) effluents^28^. Component 5 had a peak at *λ*_ex_/*λ*_em_ = 265/480 nm. Fluorescence in this region is referred to as peak A (humic-like) based on Coble^9^,^10^ and as Region V (humic acid-like) based on FRI technique by Chen, *et al.*^11^. A summary table (Table S1) lists the characteristic peaks, type classified by methods by Coble^9^,^10^ and Chen, *et al.*^11^, and the possible sources.

According to the methods for DOM fractionation developed by Coble^9^,^10^ and Chen, *et al.*^11^, DOM pool could be divided into two categories: humic-like substances and protein-like substances. Humic-like substances comprise peak A, C^9^,^10^, or Region III, V^11^. Humic-like substances are ubiquitous in almost all natural waters^9^,^10^,^29^,^30^ and are thought to originate from terrestrial organic matter from soils^31^. Humic-like fluorescence might be intensified by substantial surface runoff/lateral seepage input into ambient waterways caused by rainfall^25^. Protein-like substances comprise peak B, T_1_ and T_2_ 9,10, or Region I, II and IV^11^. Protein-like fluorescence is associated with microbially-derived organic matter^32^; hence, the presence of protein-like fluorescence could be attributed to microbially-derived organic matter originating from agricultural and rural activities involving biological processes. Protein-like substances are also found in freshwaters affected by wastewater and in productive oceanic environments^10^,^30^,^33^. Moreover, Henderson, *et al.*^34^ reported that additional peaks in protein-like region might originate from optical brightening agents used in paper brightening and household detergents which could be found in sewage-polluted waters^35^.

### Fluorescence as an indicator for NPS pollution

An approach introduced in 1980s for data mining, called SOM^36^ which is a powerful computational tool classified as artificial neural networks, was employed to explore the considerable dataset for the fluorescence properties of DOM. SOM analysis was used to assist the PARAFAC results which is an alternative to peak-picking method to discriminate between fluorescence compositions from a massive dataset.

**Figure 2:**
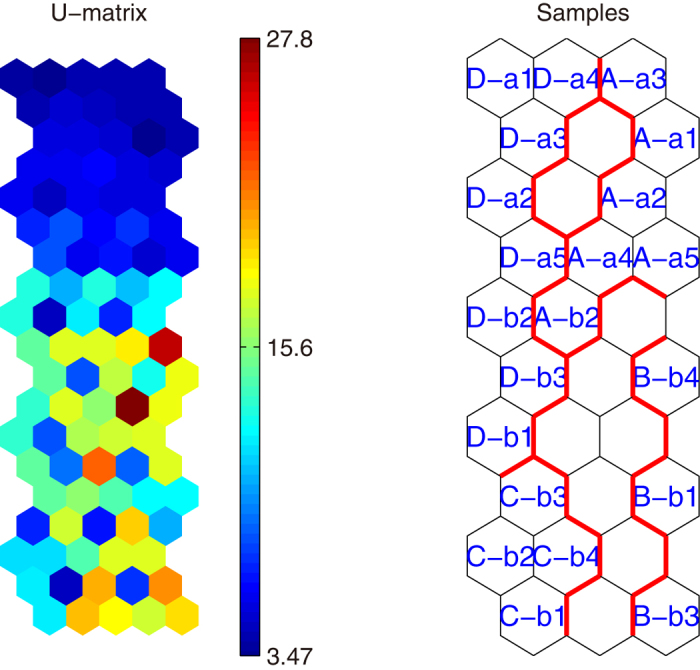
U-matrix (on left) and sample distribution map (on right) of SOM analysis. In sample distribution, “A”, “B”, “C”, “D” represent different sampling events in chronological order; “a” and “b” represent “unpolluted” and “polluted” respectively; the arabic numerals represent different sampling sites. The figures were created using MATLAB 7.0.

Sample distribution on SOM map is illustrated in Fig. 2. The SOM map is divided into two clusters according to fluorescence properties of DOM, with distinct fluorescence feature in each cluster. It is clear that the SOM map can be divided into two parts respectively in the vertical and horizontal direction. Horizontally, the SOM map can be divided into two types of water quality: the samples polluted by NPS in the bottom of the map, and the samples unpolluted in the top of the map. Compared with the samples located in the upper side of the map, the samples located in the bottom of the map consist higher content of DOM and fluorescence intensity. Vertically, the SOM map can be divided into two time periods: the samples collected in fall in the left side of the map, and the samples collected in spring and summer in the right side of the map. In spring and summer, fertilization contributed high amount of organic matter release from agricultural lands via runoff  6,25, and the rainfall intensified the organic matter input into the surrounding waterways^37^,^38^. In fall, leaching of deposited straw and litter material contributed considerable organic matter to ambient waterways^39^,^40^,^41^,^42^. From the U-matrix of Fig. 2, we can see the color is a little darker on right hand side than left hand side. Thus, we concluded the right side of the SOM map exhibits a higher DOM content and fluorescence intensity compared with the left side of the SOM map because organic matter released more in spring and summer.

**Figure 3:**
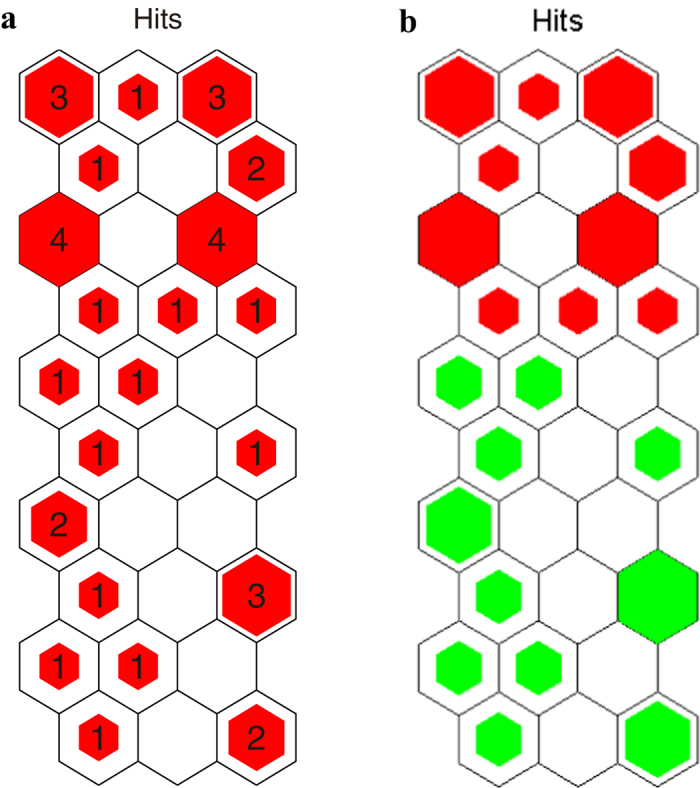
Hit histograms of SOM analysis. (**a**) the number in the neurons represents the sample number of the neuron; (**b**) red represents unpolluted samples and green represents polluted samples. The figures were created using MATLAB 7.0.

To combine the sample distribution (Fig. 2), the hit histograms were applied to illustrate how many times each neuron was the winning neuron for the dataset of water samples. Each neuron (map unit) of the hit histogram (Fig. 3) is corresponding to the neuron of the SOM map for sample distribution (Fig. 2). The difference between SOM map for sample distribution and hit histogram is that, each neuron in SOM map for sample distribution give the sample name of the most frequent best matching sample, standing for the several samples falling into this winning neuron with similar fluorescence properties, while each neuron in hit histogram gives the number of samples falling into the winning neuron. The neurons with higher number of hits represent more water samples with similar fluorescence properties. Accordingly, neurons with higher number in hit histogram reveal more typical fluorescence feature of DOM observed during the research. It can be demonstrated from Fig. 3a that the most typical map neurons (most typical fluorescence features) are located at the edges of the map. Furthermore, different colors in hit histogram reveal the difference between polluted and unpolluted water samples’ organic matter fluorescence properties. Figure 3b shows a great distinction between polluted and unpolluted water sample properties that may be indicative of a NPS pollution.

Previous studies on monitoring pollution in surface waters and drinking water supply concluded that protein-like fluorescence peaks (*e.g.* peak B and T) are the best indicators for pollution^34^ and peak C could be used as a supplementary pollution indicator^18^,^19^. Herein, a comparison between SOM analysis and peak-picking method is carried out to explore a better indicator for NPS pollution. We applied cluster analysis based on the values of peak B, T_1_, T_2_ and C to examine whether peak-picking could be considered as a better indication for NPS pollution than SOM analysis. Supplementary Fig. S1 showed that each type of water (polluted or unpolluted) could not be consistently clustered into one category, for instance, A-Pol-1 and A-Pol-3 are clustered into a class with 9 unpolluted samples in the first stage. It can be inferred that there is no consistent picked peak fluorescence character within the 15 polluted DOM or within the 21 unpolluted DOM, in terms of peak B, T and C fluorescence. Accordingly, peak-picking method could not provide a better indication for NPS pollution than SOM analysis could.

### Reliability evaluation of several Raw EEM measurements surrogate for massive EEMs under PARAFAC

**Figure 4:**
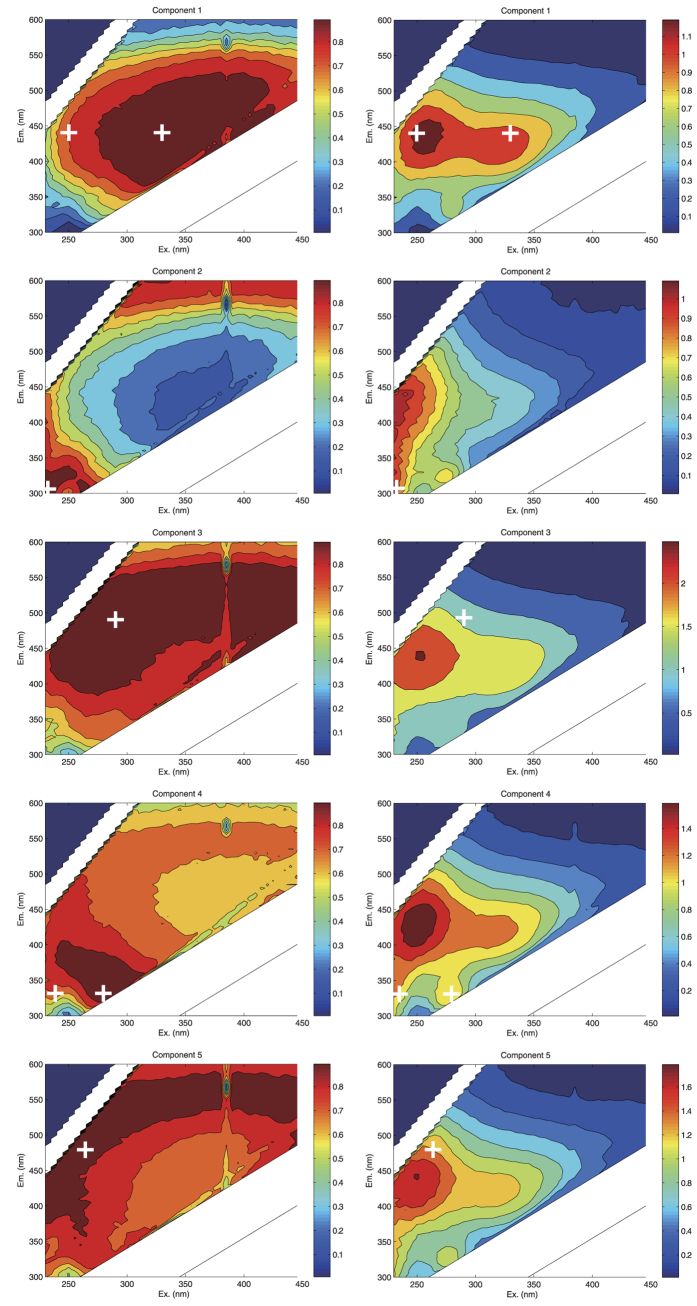
Contour plots of determination coefficients and regression coefficients for regression analysis between PARAFAC *F*_max_ and raw EEMs. White crosses in the left panels are the locations of peaks of the PARAFAC components. The figures were created using MATLAB 7.0.

To validate fluorescence components from PARAFAC as a proxy indicator for NPS pollution, the relationship between PARAFAC scores and EEM measurements was explored. Correlation between fluorescence intensities of PARAFAC component peaks and raw EEM measurements was analyzed to examine the effectiveness of fluorescence results as indicators for NPS pollution. Figure 4 shows the contour graphs of determination coefficients and regression coefficients from the regression analysis between PARAFAC intensities (*F*_max_) for component 1–5 and fluorescence intensities of each ex-em pair from original EEMs. The left panels of Fig. 4 exhibits the determination coefficients (fit of linear regression, R^2^), with the highest values (red region) indicating strongest correlations near PARAFAC component peaks (white crosses), and the relative low values (blue region) indicating poor correlations far away from PARAFAC component peaks. The right panels of Fig. 4 exhibits the regression coefficients (linear slope), with the value approaching 1.0 indicating *F*_max_ from PARAFAC is equivalent (the intercept is zero) to fluorescence intensity from original EEM measurements.

In Fig. 4, the region where the determination coefficient (R^2^) and the regression coefficient (m) are both closer to 1.0 (the intercept is zero) means more accurate and reliable prediction of fluorescence phenomenon in original EEM measurements using PARAFAC scores as proxy indicators. Additionally, the phenomenon that the reddest region is closer to the white cross in the left panels of Fig. 4 means more accurate and reliable prediction of fluorescence phenomenon in EEM measurements using PARAFAC components as proxy indicators. Accordingly, the phenomenon that R^2^ and m equivalent to 1.0 are both located at the same point, *viz*, the white cross, is the best and ideal scenario for the prediction using PARAFAC model. For component 1 in Fig. 4, the R^2^ and m at the peak point (*λ*_ex_/*λ*_em_ = 250/440 nm) and shoulder point (*λ*_ex_/*λ*_em_ = 330/440 nm) are both close to 1.0, indicating the position of component 1 peak is a good indicator for fluorescence DOM composition. For component 1 in the right panel, the region around the point that m is equivalent to 1.0 is a gentle slope, with a larger distance between two contour lines, meaning that little deviation in the fluorescence position during measurements would not significantly diminish the accuracy and reliability of prediction using PARAFAC scores as proxy indicators. However, for component 2 in the right panel, the region around the point that m is equivalent to 1.0 is a steep slope, with a small distance between two contour lines, meaning that the prediction using PARAFAC scores as proxy indicators is sensitive to the wavelength positions of EEM measurements. For component 3, the R^2^ and m near the peak point (*λ*_ex_/*λ*_em_ = 290/490 nm) are both close to 1.0, and the region encompassing the peak has a gentle slope. Accordingly, it is a good scenario to predict PARAFAC component 3 using raw EEM. For component 4, the R^2^ and m at the peak point (*λ*_ex_/*λ*_em_ = 280/330 nm) and shoulder point (*λ*_ex_/*λ*_em_ = 235/330 nm) are both close to 1.0. However, the regions around the peak and shoulder are steep slopes, meaning that the prediction is sensitive to the wavelength regions of EEMs. For component 5, the R^2^ and m near the peak (*λ*_ex_/*λ*_em_ = 265/480 nm) are also both close to 1.0, and the slope around the peak is relatively gentle. Accordingly, it is a relatively reliable for the prediction of PARAFAC component 5. From the results above, we can infer that conducting a small number of fluorescence measurements at the target excitation-emission wavelength pairs without PARAFAC analysis could still provide relatively accurate and reliable fluorescence DOM information similar to massive measurement combined with PARAFAC.

### Identification of raw EEM as proxy indicator for dissolved organic carbon (DOC) concentration

**Figure 5:**
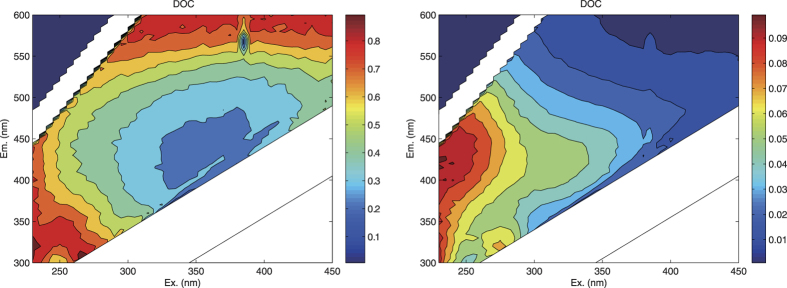
Contour plot of determination coefficients and regression coefficients for regression analysis between DOC concentrations and raw EEMs. The figures were created using MATLAB 7.0.

To verify the hypothesis that several raw EEMs could be used as surrogates for labor-intensive water quality indicators, relationship between bulk DOC concentration and raw EEM was explored. Linear correlation between DOC concentration and raw EEM measurements was analyzed to inspect the effectiveness of effortless EEM measurement as surrogate indicators to predict routine and labor-intensive water quality indicators such as DOC concentration (Fig. 5).

In Fig. 5 there exists a strong linear correlation (R^2^ > 0.8) between DOC concentration and a region of fluorescence ex-em pairs (Fig. 5). The most reliable prediction namely highest R^2^ value (R^2^ > 0.8) was located within excitation 230 to 285 nm and emission 305 to 455 nm of EEM region. This region includes peak B, which was associated with tyrosine-like, and PARAFAC component 2. In the last section discussing reliability evaluation of Raw EEM measurements surrogate for PARAFAC EEMs, there is a strong correlation between raw EEMs and scores of PARAFAC component 2 in the region encompassing the peak of component 2. Accordingly, we can infer that there might be a significant correlation between DOC concentrations and scores of PARAFAC component 2.

Using optical properties as surrogates for labor-intensive routine water quality indicators has been studied for many years^10^,^25^. Absorption coefficients from absorption spectrum (*e.g.* ultraviolet absorbance at 254 nm (UVA_254_)) and fluorescence values from EEM spectrum (*e.g.* FDOM_370/460_) have been shown to be reliable predictors of DOC concentration^43^,^44^,^45^. However, the determination coefficient between UVA_254_ and DOC in this study (Fig. S2) did not show a better fit than the correlation between EEM and DOC (Fig. 5). Here, R^2^ value is 0.70 for correlation between UVA_254_ and DOC, lower than that between DOC and a region within raw EEM (excitation 230 to 285 nm and emission 305 to 455 nm) (Fig. 5, Fig. S2), and that between DOC and PARAFAC component 2 or 5 (Table 1). Moreover, the correlation between UVA_254_ and DOC concentrations was carbon source dependent so that different carbon source will show a different slope of linear regression. Distinct linear regressions between UVA_254_ and DOC concentrations imply that different carbon sources have different chemical characteristics. The slope of the linear regressions between UVA_254_ and DOC concentrations is considered as specific ultraviolet absorbance at 254 nm (SUVA_254_). SUVA_254_, in general, is proportional to the aromaticity of DOC (the amount of chromophore or aromatic carbon per unit of DOC) and has also been widely considered as a surrogate for indicating DBP precursors^8^,^46^. From the view of mechanism, a low SUVA_254_ value for DOC indicates that few conjugated double bonds and aromatic carbon existed per unit DOC. In addition, using one fixed fluorescence peak value (*e.g.* FDOM_370/460_) will bring bias to the prediction of DOC concentration, because the best wavelength location for fluorescence peak value to predict DOC will vary with different conditions (*e.g.* DOM source). In this dataset, the best DOC prediction location falls on *λ*_ex_/*λ*_em_ = 265/310 nm, both of which emission and excitation wavelengths were shifted towards shorter wavelengths away from FDOM location. Therefore, fluorescence peaks used to predict DOC concentration or other water quality indicators are DOM source dependent and should not be fixed to several single EEM locations.

### Relationship between DOC concentration and PARAFAC components

As mentioned above, 5 fluorescence components were obtained from PARAFAC. We further inspected the relationship between DOC concentration and PARAFAC components with SOM approach.

**Figure 6:**
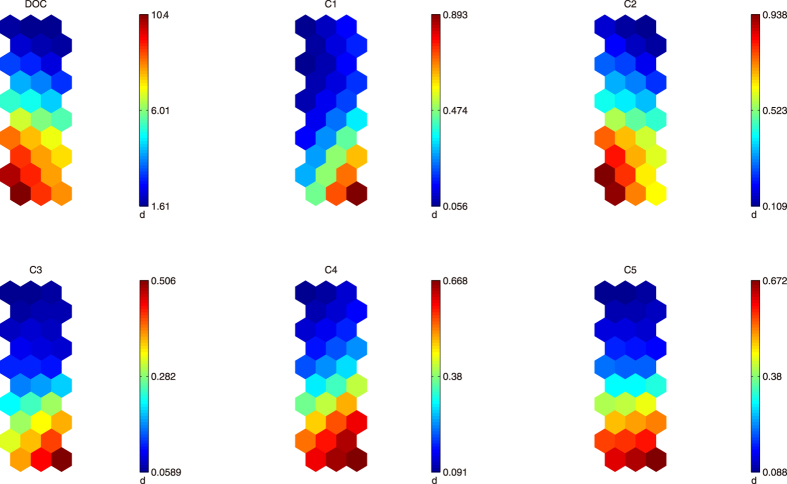
Abstract visualization of the relationships between DOC concentration (mg L^−1^) and fluorescence values (Raman unit) of 5 PARAFAC components. The figures were created using MATLAB 7.0.

The component planes for each variable of the SOM output are illustrated in Fig. 6. The component planes of the same clusters have a certain similarity, that is, if corresponding neurons’ color trends are similar, there is a certain correlation between them. Results suggested that high DOC concentrations (>6.01 mg L^−1^) are a response of high PARAFAC component 1 scores (>0.474 Raman unit (RU)), high PARAFAC component 2 scores (>0.523 RU), high PARAFAC component 3 scores (>0.282 RU), high PARAFAC component 4 scores (>0.380 RU), and high PARAFAC component 5 scores (>0.380 RU), collectively (Fig. 6). Regression analysis indicated there were significant linear correlations between DOC concentration and the five PARAFAC components, and component 2 gives the best prediction (R^2^ = 0.87). Incorporation of all the five components into the model resulted in a better fit (R^2^ = 0.91) (Table 1), suggesting that each of the five components contributed a part of the DOM to the bulk DOC, despite a weak correlation (R^2^ = 0.19) between component 1 and DOC concentration.

The strongest relationship between DOC concentration and PARAFAC component 2 indicated that aromatic protein associated with peak B (tyrosine-like) contributed the greatest part to the bulk DOC. Since aromatic protein is autochthonous (microbially derived) DOM, it can be inferred that anthropogenic practice such as agricultural and rural NPS pollution contributed high content of autochthonous DOM. NPS pollution from agricultural lands via runoff or seepage contained soluble microbial products formed in the biochemical processes in agricultural fields (*e.g.* paddy fields), which could be a source of aromatic protein in DOM in samples. The aromatic protein is also known as a kind of DBP precursors^47^.

## Methods

### Site Description

Sampling sites were located in a small watershed (119°71′E, 30°46′N) in Quanchengwu Village Luniao Town Yuhang District, Hangzhou, Zhejiang. The annual average temperature was 17.5 °C, with a summer average temperature of 16.2 °C and a winter average temperature of 3.8 °C. The annual rainfall is 1454 mm and annual average relative humidity is 70.3%. This watershed is the origin of East Tiaoxi River. The water of the watershed originated from the hills within it, with a good closure, thus the watershed was a proper site to study the effect of NPS pollution.

### Sampling and Analyses

**Figure 7:**
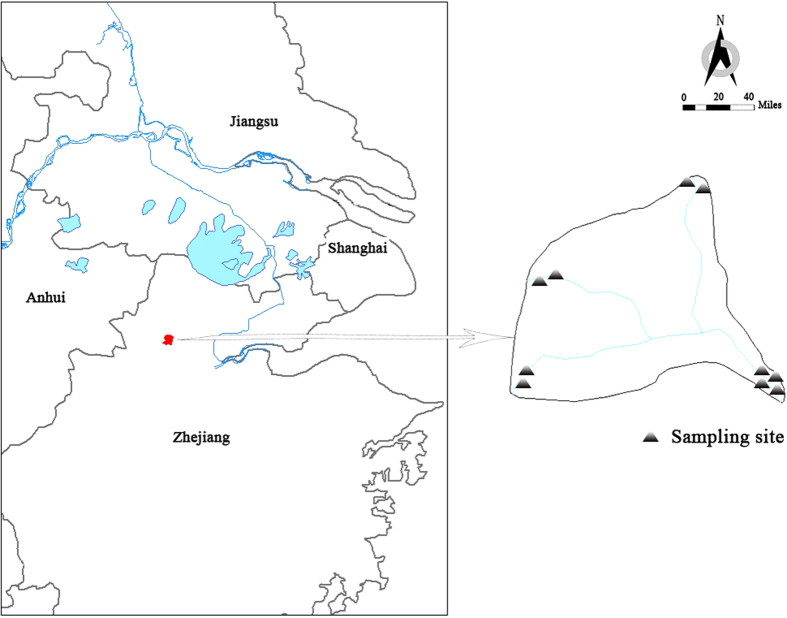
Location of sampling sites for the watershed in Quanchengwu Village, Luniao Town, Yuhang District, Hangzhou, Zhejiang. The maps were created using ArcGIS 10.1.

To assess the effects of NPS pollution on water quality, samples were collected from six sites in the upstream of river and from four sites in the downstream of river over the whole year of 2014 (Fig. 7). The sampling dates were Apr 22, Jun 17, Sep 5 and Nov 2 respectively. Samples were collected over a 1-d period according to a synoptic sampling approach. A combination of depth integrating sampling and grab sampling was employed to collect river samples. As to unsafe sites, grab sampling was chosen. The river was well mixed due to high gradient and lack of point sources, so grab sampling was acceptable. Whole water samples were collected in polyethylene terephthalate (PET) bottles. Samples were 50 mL triplicates extracted in the laboratory from a 3 L sample. Samples were kept on ice and in the dark. Dissolved analytes were analyzed from samples filtered through precombusted 60-mm, 0.45-μm nominal pore size GF/F filters. Laboratory experiments indicated no fluorescent leachates from the PET bottles during this period.

DOC concentration was determined with a MultiN/C2100TOC/TN analyzer of analytikjenaAG with a detection limit of 0.05 mg L^−1^. Fluorescence EEMs were measured on filtered samples with an F-4500 fluorescence spectrophotometer (Hitachi, Shanghai) with a 5-nm band pass and 0.050-s integration time. Fluorescence intensity was measured at excitation wavelengths of 230 to 450 nm at 5-nm intervals and emission wavelengths of 300 to 600 at 5-nm intervals on room temperature samples (25 °C) in a 1-cm quartz cell. Inner filter corrections were applied to EEMs with ultraviolet absorbance at 254 nm (UVA_254_) greater than 0.03 (1-cm cuvette) as described by Gu and Kenny^48^.

### Data Analysis

#### SOM approach

To visualize the cluster of sample distribution and the relationships between DOM bulk indicators and PARAFAC components, the SOM approach was performed with MATLAB (Version 7.00) software. The SOM is a competitive artificial neural networks based on unsupervised learning^49^, which requires merely SOM toolbox and some basic functions to achieve its function in MATLAB. The principle of SOM analysis can be found in many studies^50^,^51^. In this study, we developed two datasets to serve two objectives. Firstly, a dataset with a 36 × 1748 matrix was established, comprising 36 data samples and 1748 ex-em pairs as variables, in order to visualize the distribution and cluster of samples based on fluorescence properties. Secondly, a dataset with a 36 × 6 matrix was established, comprising 36 data samples and 6 variables including DOC concentration and five PARAFAC components’ scores. For the first purpose, three-dimensional EEM of 36 samples were unfolded to two-dimensional vectors, where each row represents data sample and each column represents unfolded ex-em pairs. The sample distribution of SOM map and hit histograms were obtained for clustering of samples. For the second purpose, a series of component planes was obtained for visualization of correlation analysis. In the training section of SOM running, each neuron of input layer of SOM is associated with all input samples and has reference vector with SOM weights. The neuron weights were processed with linear initialization along the two greatest eigenvectors of the input matrix^36^. The ultimate size (10 × 3) of output SOM map was determined by the ratio of the two greatest eigenvalues of the input matrix. The output U-matrix visualized the distances between two map neurons, where the reddest U-matrix map units represent the border of clusters. The output component planes visualized the property distribution of samples, where similar component patterns indicate positive correlations.

#### PARAFAC analysis

To decompose the fluorescence signal into underlying individual fluorescence composition information, the PARAFAC analysis was performed with MATLAB (Version 7.00) software. PARAFAC analysis is a competitive technique for modeling and visualizing complicated multi-variate data^52^, which requires merely certain toolboxes and some basic functions to achieve its function in MATLAB. The basic principle of PARAFAC analysis is an alternating least-squares algorithm which decomposes the data into a set of trilinear terms and a residual array, and it can be found in many studies^20^,^52^.

PARAFAC model was derived for all samples using DOMFluor Toolbox for MATLAB with non-negativity constraints applied on all modes. The majority of Raman scatter was removed by subtracting the pure water spectrum from the sample spectrum. The first and second order scatter peaks were cut from EEM spectra and replaced with zeros. Two different split half analyses were run to inspect whether the model was validated. Tucker congruence coefficients^53^ were used for comparing components between different PARAFAC models. Finally, a validated and fitted model was obtained, and a dataset comprising the fluorescence intensities of each component in each sample and the emission and excitation loadings of each component was exported.

To evaluate the potential for estimating DOC concentrations and PARAFAC scores from raw EEMs, the original measured EEM data were regressed against the DOC concentrations the maximum fluorescence (*F*_max_) of each component obtained via PARAFAC. To each ex-em wavelength pair, we can get a 36 × 1 vector of raw EEM fluorescence intensities. This 36 × 1 vector was regressed against the 36 × 1 vector of DOC concentrations and 36 × 1 vector of PARAFAC scores of each component. Thus, regression coefficients (m) and determination coefficients (R^2^) were obtained as a function of wavelength, which can be plotted as contour graphs.

## Additional Information

**How to cite this article**: Zhang, Y. *et al.* A novel approach combining self-organizing map and parallel factor analysis for monitoring water quality of watersheds under non-point source pollution. *Sci. Rep.* **5**, 16079; doi: 10.1038/srep16079 (2015).

## Supplementary Material

Supplementary Information

## Figures and Tables

**Table 1:** Regression analysis between DOC concentration and PARAFAC components.

		C 1	C 2	C 3	C 4	C 5	C 1–5
DOC	R^2^	0.19	0.87	0.53	0.60	0.72	0.905
	m	4.824	10.154	14.859	11.530	12.501	/
	P value	0.009	<0.001	<0.001	<0.001	<0.001	<0.001

R^2^ means determination coefficient, namely fit of linear regression, and m means regression coefficient, namely linear slope.
