# Supplementary material for: A novel approach combining self-organizing map and parallel factor analysis for monitoring water quality of watersheds under non-point source pollution
Source: Sci Rep. 2015 Nov 3;5:16079. doi: 10.1038/srep16079 (PMC4630639; doi:10.1038/srep16079)
Supplement: Supplementary Information [file srep16079-s1.pdf]

Supplementary Information

A novel approach combining self-organizing map and parallel factor analysis for monitoring water quality of watersheds under non-point source pollution

Yixiang Zhang[1], Xinqiang Liang[1, 2, *], Zhibo Wang[1] & Lixian Xu[1]

[1]College of Environmental and Resource Sciences, Zhejiang University, Hangzhou 310058, China.

[2]Zhejiang Provincial Key Laboratory for Water Pollution Control and Environmental Safety.

Correspondence and requests for materials should be addressed to X.L. (Phone: +86-571-88982018; Email: liang410@zju.edu.cn)

Figure legends

Fig. S1 Cluster result based on fluorescence values of peak A, T_1, T_2, B. “A”, “B”, “C”, “D” represent different sampling events in chronological order; “Unp” and “Pol” represent “unpolluted” and “polluted” respectively; the arabic numerals on the vertical axis represent different sampling sites. The figure was created using SPSS 20.

Fig. S2 Linear regression between DOC concentration and UVA_{254} (n = 36). The figure was created using OriginPro 8.

Table S1 A summary of 5 PARAFAC components, their classification and possible sources.

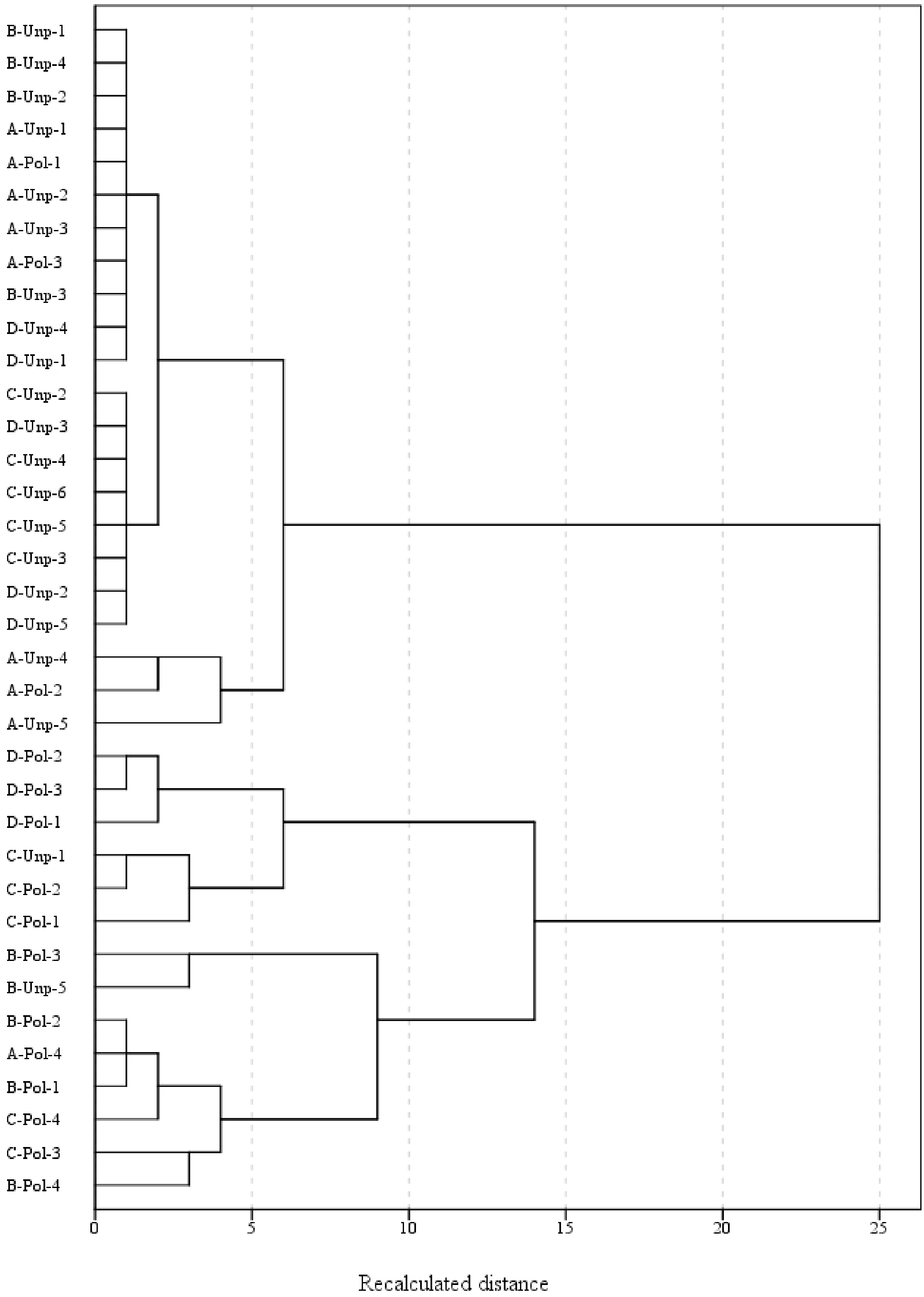

Fig. S1 Cluster result based on fluorescence values of peak A, T_1, T_2, B. “A”, “B”, “C”, “D” represent different sampling events in chronological order; “Unp” and “Pol” represent “unpolluted” and “polluted” respectively; the arabic numerals on the vertical axis represent different sampling sites.

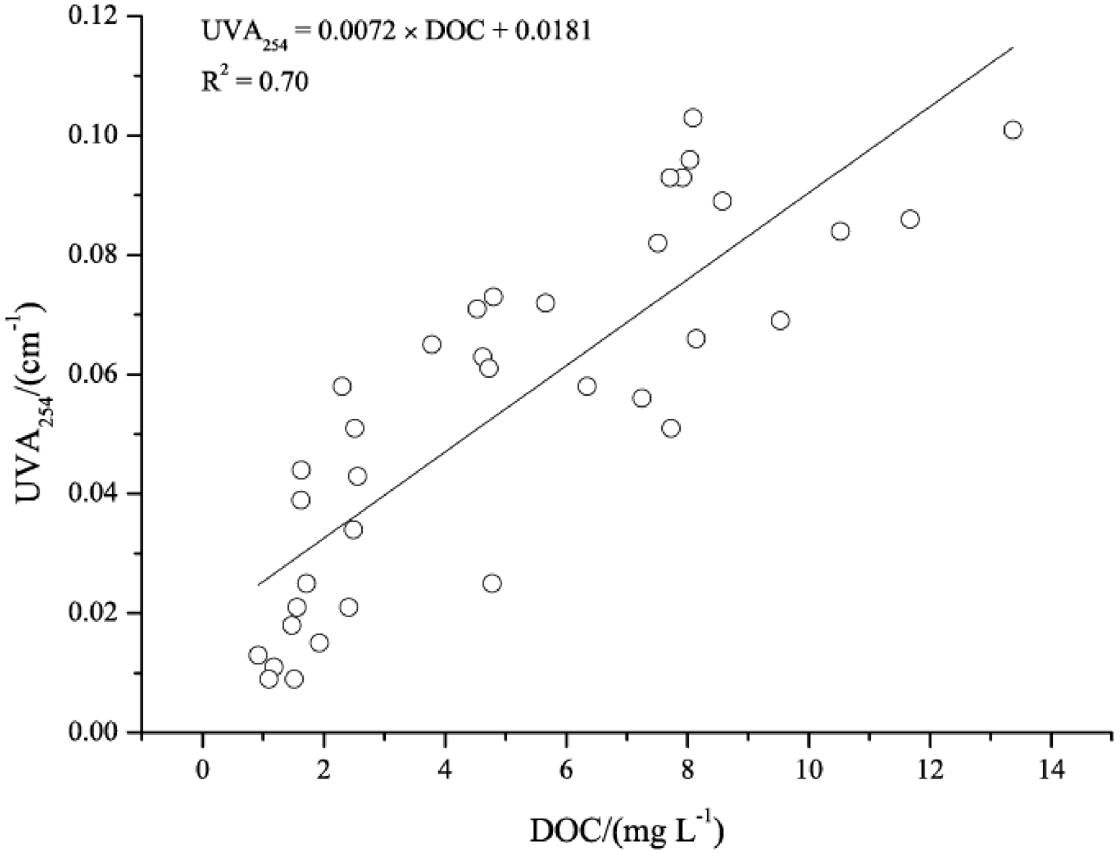

Fig. S2 Linear regression between DOC concentration and UVA_{254} (n = 36).

Table S1 A summary of 5 PARAFAC components and their classification.

PARAFAC component	Component 1	Component 2	Component 3	Component 4	Component 5
Peak/Shoulder location ($\lambda_{ex}/\lambda_{em}$)	250/440 nm (peak) 330/440 nm (shoulder)	230/300 nm (peak)	290/490 nm (peak)	280/330 nm (peak) 235/330 nm (shoulder)	265/480 nm (peak)
Peak-picking classified by Coble [1,2]	Peak A (humic-like)	Peak B (tyrosine-like)	Peak C (humic-like)	Peak T_1 (tryptophan-like)	Peak A (humic-like)
FRI classified by Chen, *et al.* [3]	Region III (fulvic acid-like)	Region I (aromatic protein)	Region V (humic acid-like)	Region IV (soluble microbial product (SMP)-like)	Region V (humic acid-like)
Possible sources	humic, terrestrial, allochthonous [2,4,5]	Autochthonous [2,4,5]	terrestrial, anthropogenic, agriculture [2,4,5]	Autochthonous [2,4,5]	humic, terrestrial, allochthonous [2,4,5]

References:

1. Coble, P. G. Characterization of marine and terrestrial DOM in seawater using excitation-emission matrix spectroscopy. *Mar. Chem.* **51,** 325-346 (1996).

2. Coble, P. G. Marine optical biogeochemistry: the chemistry of ocean color. *Chem. Rev.* **107,** 402-418 (2007).

3. Chen, W., Westerhoff, P., Leenheer, J. A. & Booksh, K. Fluorescence excitation-emission matrix regional integration to quantify spectra for dissolved organic matter. *Environ. Sci. Technol.* **37,** 5701-5710 (2003).

4. Stedmon, C. A., Markager, S. & Bro, R. Tracing dissolved organic matter in aquatic environments using a new approach to fluorescence spectroscopy. *Mar. Chem.* **82,** 239-254 (2003).

5. Stedmon, C. A. & Markager, S. Resolving the variability in dissolved organic matter fluorescence in a temperate estuary and its catchment using PARAFAC analysis. *Limnol. Oceanogr.* **50,** 686-697 (2005).
